# Prognostic Significance of p27 and Survivin in *H. pylori *Gastritis and Gastric Cancer 

**DOI:** 10.31557/APJCP.2021.22.11.3553

**Published:** 2021-11

**Authors:** Noha Said Helal, Zeinab Omran, Tarek Aboushousha, Magdy Youssef, Afkar Badawy, Mohammed A Aboul-Ezz, Mona Moussa

**Affiliations:** 1 *Department of Pathology, Theodor Bilharz Research Institute, Giza, Egypt. *; 2 *Department of Hepatology and Gastroenterology, Theodor Bilharz Research Institute, Giza, Egypt. *

**Keywords:** Immunohistochemistry- H. pylori, intestinal metaplasia, gastric cancer

## Abstract

**Objective:**

to assess expression of p27 and survivin in chronic gastritis with/without *H. pylori *± intestinal metaplasia (IM) and in intestinal-type gastric cancer (IGC).

**Materials and Methods:**

Immunohistochemical staining for p27 and survivin on paraffin-embedded sections of 20 chronic gastritis, 20 *H. pylori *gastritis, 15 *H. pylori *gastritis with IM, 50 IGC, and 10 controls. Positivity (number of positive cases) and expression (mean percentage of positive gastric cells) for both proteins were evaluated.

**Results:**

P27 positivity and expression decreased from control to chronic gastritis to *H. pylori *gastritis to *H. pylori *gastritis with IM. In IGC, p27 positivity and expression were lower than controls and chronic gastritis but higher than *H. pylori *gastritis ±IM. High grade and advanced stage IGCs have insignificantly lower p27 positivity and expression than low grade and early stage IGCs. By contrast, survivin positivity and expression increased from chronic gastritis to *H. pylori *gastritis to *H. pylori *gastritis with IM to IGCs. High grade and advanced stage IGCs have significantly higher survivin positivity and expression than low grade and early stage IGCs. Males have higher positivity and expression for p27 and survivin than females.

**Conclusion:**

Inverse relation between p27 and survivin in *H. pylori *gastritis, *H. pylori *gastritis with IM and IGCs lesions, suggesting that both proteins could be used as potential prognostic and/or diagnostic biomarkers in *H. pylori *and IM associated- gastritis as well as in IGC.

## Introduction

*Helicobacter pylori* (*H. Pylori*) colonize gastric mucosa and contribute to the development of chronic gastritis that progresses to atrophic gastritis, intestinal metaplasia (IM), dysplasia, and eventually gastric cancer (GC) (Rugge et al., 2017). Gastric IM is an established early step in gastric carcinogenesis affecting about 30% of the individuals infected with *H. pylori *(Camilo et al., 2012). Gastric cancer (GC) ranks fifth for incidence and fourth for mortality worldwide (Sung et al., 2021). Despite recent advances in surgical techniques and adjuvant therapy after surgery, patients with gastric cancer are often given a poor prognosis due to the difficulty of early diagnosis and high recurrence rate (Kanagavel et al., 2015). Therefore, it is of great importance to find new molecular markers that can help evaluate the prognosis or develop novel therapies for gastric cancer (Sun et al., 2016).

P27 is a cyclin-dependent kinase inhibitor that regulates cell cycle progression through inhibition of the G2/M phase followed by apoptosis of cells and induces a block of the cell cycle thus maintaining cells in the resting (G0) state (Yanagi et al., 2017). Therefore, its defective regulation can lead to uncontrolled proliferation of cells (Chu et al., 2008). P27 also acts as a tumor suppressor gene and is involved in the regulation of differentiation, drug resistance, cell migration, and in the response to inflammatory stimuli (Sun et al., 2016). Most reports supported the role of p27 in promoting apoptosis (Supriatno et al., 2002 and Eguchi et al., 2004), meanwhile, others suggested the antiapoptotic effect of p27 (Yang et al., 2000 and Philipp-Staheli et al., 2001).

Survivin, a member of the Inhibitor of Apoptosis Protein (IAP) family, is expressed highly at G2/M phase and declines rapidly in G1 phase of cell cycle. It is an antiapoptotic protein, that regulates cell survival, reduces the cell loss rate, and promotes both cell proliferation and providing advantage to a rapidly growing tumor and consequently to a neoplastic transformation (Duffy et al., 2007). It can serve as a universal tumor antigen because it is expressed in most human malignancies and has the potential to trigger immune effector responses. So, blocking its function by immunotherapeutic or molecular approaches could be a promising therapeutic strategy in cancer (Garg et al., 2016).

This work aims to assess immunoreactivity of p27 and survivin in chronic gastritis, gastric precancerous lesions (*H. pylori *gastritis and intestinal metaplasia), and intestinal-type gastric cancer, to assess their role as prognostic indicators of progression of precancerous lesions to gastric cancer. 

## Materials and Methods

*Samples*

This retrospective study included formalin-fixed paraffin-embedded blocks of 115 gastric specimens included: 10 blocks of cases with minimal gastritis, negative for H. pylori, IM, and dysplasia (served as controls); 55 blocks of chronic gastritis specimens divided as 20 chronic gastritis, 20 *H. pylori *gastritis, and 15 *H. pylori *gastritis associated with IM, as well as 50 intestinal-type gastric cancer (IGC) blocks. All blocks were collected from the Pathology Department, TBRI, in a period from 2019-2020. Specimens of chronic gastritis were obtained as endoscopic biopsies and specimens of IGC were obtained as partial/total gastrectomy specimens. 

The study protocol was approved by the Institutional Review Board of TBRI, for the protection of human subjects and adopted by the 18th world medical assembly, Helsinki, Finland (2013).

*Histopathological technique and evaluation*

Paraffin-embedded sections were cut in a thickness of 4 µm, stained by hematoxylin and eosin (H&E) stain for routine histopathological examination and diagnosis. They were also stained by Giemsa stain for detection of H. pylori. Chronic gastritis sections were evaluated for intensity of inflammation, presence/absence of H. pylori, IM, and dysplasia. Sections of IGC were examined for tumor grading and staging according to the international histological classification proposed by the World Health Organization (WHO), 2019. Gastric cancer is considered as low grade (well-differentiated) or high grade (moderately or poorly differentiated) and is considered as either early (pT1) or advanced (≥pT2) (Cameiro et al., 2019). Studied gastric cancer lesions were located at antrum (41/50) or corpus/funds (9/50).

*Immunohistochemical (IHC) technique*

The sections were put in the oven at 60°C for 4 hours, deparaffinized in xylene, rehydrated in a graded ethanol series, and treated with 3% hydrogen peroxide solution for 10 min. Antigen retrieval was done by microwaving the tissue in 10 mmol/L citric acid buffer for 12 min, then cooling at room temperature for 2 h. The sections were incubated with P27: (F-8) (sc-1641, Santa Cruz Biotechnology, Santa Cruz, CA, USA) and Survivin (code M3624, Dako, Copenhagen, Denmark), at dilution of 1:100 for overnight at 4°C. Sections were then washed three times for 5 min in PBS. Non-specific staining was blocked with 0.5% casein and 5% normal serum for 30 min at room temperature. Finally, staining was developed with diaminobenzidine substrate, and sections were counterstained with hematoxylin, dehydrated with graded ethanols, and mounted. For each setting; negative controls were carried out in which phosphate-buffered saline was used instead of the primary antibody. Positive control was lymphocyte in the same specimen as an internal control for P27 and duodenal tissue for Survivin.

*Assessment of Immunostaining*

The sections were examined by using light microscope (Scope A1, Axio, Zeiss, Germany). Photomicrographs were taken using a microscope camera (AxioCam, MRc5, Zeiss, Germany). Two experienced pathologists independently examined the immunoreactivity of p27 and survivin while blind to the clinicopathologic data of patients. At least 10 high-power fields at 400 x magnification were chosen randomly for each section. Cases with >5% positive gastric/tumor cells in the section were regarded as positive expression. Both positivity (number of positive cases) and expression (mean percentage of positively stained gastric cells) were evaluated.

*Statistical analysis*

Analyses were performed using SPSS version 23 (IBM Corp., Armonk, New York, USA). The significance of differences in means was calculated using One-way Anova or T-test when indicated. Fischer’s exact test was used to assess the significance of differences in clinicopathological characteristics across categories of p27 and survivin expression. Comparison of the difference in percentage between groups was evaluated using two-tailed Spearman test. Differences were considered statistically significant whenever the p values were <0.05.

## Results

*P27 Immunoreactivity*

**Figure 1:**
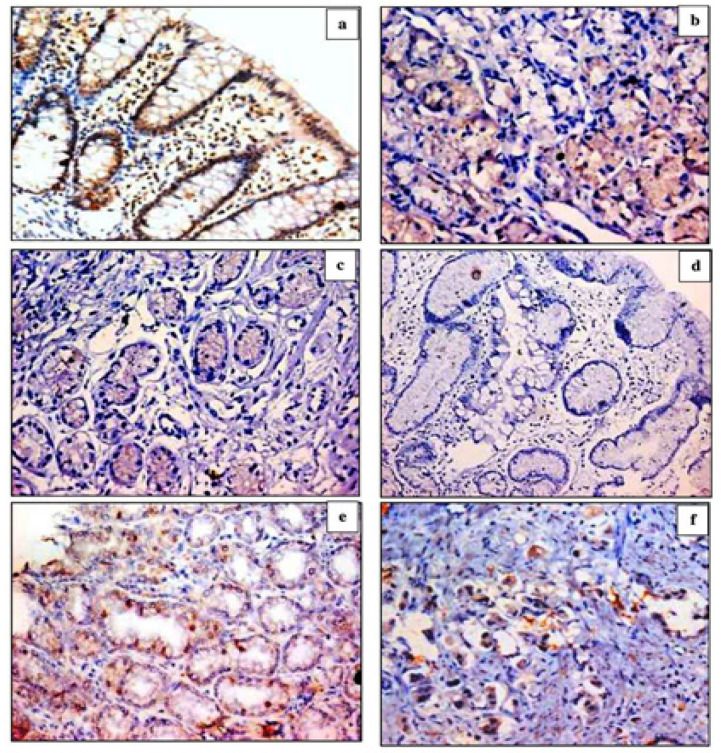
Immunohistochemical Sstaining of p27 (a) Control case with positive expression for p27 in the cytoplasm of gastric mucosal cells and nuclei of lymphocytes (x400), (b) Chronic gastritis with cytoplasmic expression for p27 in ~ 40% of gastric mucosal cells (x400), (c) H. pylori gastritis with cytoplasmic expression for p27 in ~ 25% of gastric mucosal cells (x400), (d) H. pylori gastritis with intestinal metaplasia, negative for p27 (x400), (e) Low grade intestinal-type gastric cancer with nuclear expression for p27 in ~40% of malignant gastric cells (x400), (f) High grade intestinal-type gastric expression with cytoplasmic and nuclear expression for p27 in ~25% of malignant gastric cells (x400)

We found p27 immunoreactivity in the nucleus and/or cytoplasm of gastric cells and also in the lymphocytes located in lamina propria, which were served as an internal control. We observed nuclear p27 staining in controls, cytoplasmic staining in chronic gastritis lesions with/without *H. pylori *and IM, while nuclear/cytoplasmic staining in IGC lesions. We detected p27 positivity in 5/10 (50%) of controls, 5/20 (25%) of chronic gastritis, 4/20 (20%) of *H. pylori *gastritis, and 2/15 (13%) of *H. pylori *gastritis with IM, without statistically significant difference between these groups. P27 expression was gradually decreased from chronic gastritis to *H. pylori *gastritis, to *H. pylori *gastritis with IM, with a statistical difference between *H. pylori *gastritis and *H. pylori *gastritis with IM compared to controls (Table 1) (Figure 1). In IGC cases; positivity and expression of p27 decreased compared to controls and chronic gastritis. High-grade and advanced-stage IGCs showed reduced p27 positivity and expression compared to low-grade and early-stage cancers without statistical significance (Table 1) (Figure 1). By Spearman correlation, p27 positivity and expression showed no significant correlation with grade of differentiation (p=0.710, 0.510 respectively) or stage of invasiveness (p=0.490, 0.360 respectively). 

*Survivin Immunoreactivity*

**Figure 2:**
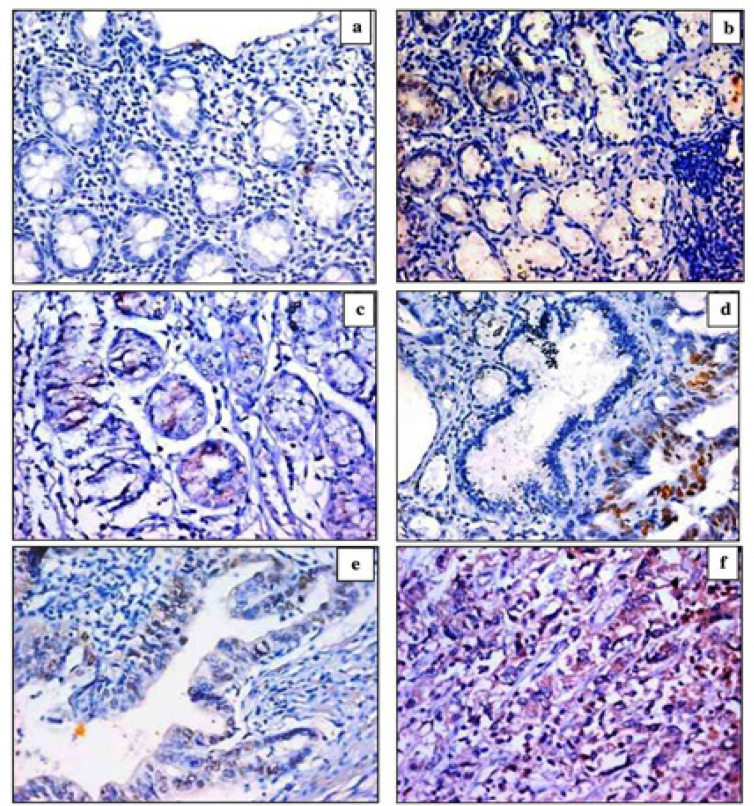
Immunohistochemical Staining of Survivin (a) Control, negative for survivin, (b) Chronic gastritis with nuclear expression for survivin in ~10% of gastric mucosal cells (x400), (c) *H. pylori* gastritis with cytoplasmic expression for survivin in ~10% of gastric mucosal cells (x400), (d) H. pylori gastritis with intestinal metaplasia with nuclear expression for survivin in ~10% of gastric mucosal cells (x400), (e) Low grade intestinal-type gastric cancer with nuclear and cytoplasmic expression for survivin in ~20^% of malignant gastric cells (x400), (f) High grade intestinal-type gastric cancer with cytoplasmic expression for survivin in ~70^% of malignant gastric cells (x400)

We found survivin immunoreactivity in the nucleus and/or cytoplasm of gastric cells. Nuclear expression was observed mainly in chronic gastritis, cytoplasmic expression in H. pylori-associated lesions (*H. pylori *gastritis ± IM), and both nuclear and cytoplasmic expressions in IGC lesions. All control cases were negative for Survivin expression. Survivin positivity was seen in 2/20 (10%) of chronic gastritis, 3/20 (15%) of *H. pylori *gastritis, 3/15 (20%) of *H. pylori *gastritis with IM, and 13/50 (26%) of IGC cases. Survivin expression increased from chronic gastritis to *H. pylori *gastritis, to *H. pylori *gastritis with IM, then significantly increased in IGC cases (Figure 2) (Table 2). In IGC cases; survivin positivity and expression increased significantly in high-grade and advanced-stage cancers compared with low-grade and early-stage ones (Table 2). By Spearman correlation test, survivin positivity and expression were significantly correlated with male sex (p=0.008 and p=0.021 respectively), grade of differentiation (p=0.007 and p=0.001 respectively) and stage of invasiveness (p=0.000 and p=0.000 respectively). 

Table (3) shows that males had higher p27 and survivin positivity and expression than females in all studied groups.

**Table 1:** P27 Immunoreactivity in the Studied Groups

			P27 immunopositivity			P27 expression (% of positive gastric cells)	
			Positive	Negative	P value	Mean ± SD	P value
Groups (N.)			N. (%)	N. (%)			
Control (10)			5 (50)	5 (50)		19.00 ± 20.79	
Chronic Gastritis (20)			5 (25)	15 (75)	p>0.05*	12.25 ± 22.27	p>0.05*
*H. Pylori *gastritis (20)			4 (20)	16 (80)	p>0.05*	4.50 ± 9.30	P<0.05*
*H. pylori* gastritis with IM (15)			2 (13.3)	13 (86.7)	p>0.05*	2.33 ± 6.23	P<0.05*
IGC (50)			11 (22)	39 (78)	p>0.05*	10.50 ± 20.61	p>0.05*
	Grade of differentiation	Low Grade (34)	8 (23.5)	26 (76.5)	p>0.05	12.50 ± 23.10	p>0.05
		High Grade (16)	3 (18.8)	13 (81.2)		6.25 ±13.60	
	Stage of invasion	Early Stage (37)	9 (24.3)	28 (75.7)	p>0.05	12.57 ± 27.78	p>0.05
		Advanced Stage (13)	2 (15.4)	11 (84.6)		4.61 ± 11.27	

N, number; *H. Pylori*, Helicobacter Pylori; IM, Intestinal Metaplasia; IGC, Intestinal-type gastric cancer; *, Compared to controls

**Table 2:** Survivin Immunoreactivity in the Studied Groups

			Survivin immunopositivity			Survivin expression (% of positive gastric cells)	
			Positive	Negative	P value	Mean ± SD	P value
Groups (N.)			N. (%)	N. (%)			
Control (10)			0	10 (100)		0.00 ± 0.00	
Chronic Gastritis (20)			2 (10)	18 (90)	p>0.05*	5.71 ± 9.76	p>0.05*
*H. Pylori* gastritis (20)			3 (15)	17 (85)	p>0.05*	6.25 ± 15.28	p>0.05*
*H. pylori* gastritis with IM (15)			3 (20)	12 (80)	p>0.05*	8.00 ± 16.56	p>0.05*
IGC (50)			13 (26)	37 (74)	p>0.05*	16.00 ± 28.93	P<0.05*
	Grade of differentiation	Low Grade (34)	5 (14.7)	29 (85.3)	P<0.01	5.88 ± 15.00	P<0.001
		High Grade (16)	8 (50)	8 (50)		37.5 ±39.90	
	Stage of invasion	Early Stage (37)	5 (13.5)	32 (86.4)	P<0.01	5.04 ± 14.45	P<0.001
		Advanced Stage (13)	8 (61.5)	5 (38.5)		48.15 ± 38.20	

N, number; *H. pylori*, Helicobacter pylori; IM, intestinal metaplasia; IGC, intestinal-type gastric cancer; *, Compared to control

**Table 3:** Sex Distribution in p27 and Survivin Positive Cases

	Positive p27 expression				Positive survivin expression			
Diagnosis (N.)	No. of +ve cases	Male N. (%)	Female N. (%)	P value	No. of +ve cases	Male N. (%)	Female N. (%)	P value
		(mean±SD)	(mean±SD)			(mean±SD)	(mean±SD)	
Control (10)	5	5 (100)	0	p>0.05	0	0	0	0
		(23.75 ± 20.66)	0	p>0.05				
Chronic Gastritis (20)	5	3 (60)	2 (40)	p>0.05	2	2 (100)	0	p>0.05
		(16.11 ± 24.72)	(9.09 ± 20.71)	p>0.05		(3.64 ± 8.09)	0	p>0.05
*H. Pylori* gastritis (20)	4	4 (100)	0	p>0.05	3	3 (100)	0	p>0.05
		(8.18 ± 11.46)	0	P<0.05		(11.36 ± 19.51)		p>0.05
*H. pylori* gastritis with IM (15)	2	2 (100)	0	p>0.05	3	3 (100)	0	p>0.05
		(3.50 ± 7.47)	0	p>0.05		(12.00 ± 19.31)	0	p>0.05
IGC (50)	11	8 (72.7)	3 (27.3)	P<0.05	13	10 (77)	3 (23)	p>0.05
		(13.75 ± 20.24)	(2.63 ± 11.55)	P<0.01		(19.66 ± 29.82)	(10.95 ± 27.56)	p>0.05
Total (115)	27	17 (63)	10 (37)	p>0.05	21	18 (86)	3 (14)	P<0.01
		(12.90 ± 20.44)	(3.17 ± 11.92)	P<0.01		(13.75 ± 23.87)	(5.90 ± 20.74)	p>0.05

N, number; +ve, positive; *H. pylori*, Helicobacter pylori; IM, intestinal metaplasia; IGC, intestinal-type gastric cancer

## Discussion

Chronic infection with *H. pylori *is the strongest risk factor for gastric cancer (Cabebe, 2020) and IM is a well-recognized pre-malignant gastric lesion, which is closely linked to *H. pylori *infection (Zali et al., 2011). In this study, we analyzed, by IHC, the immunoreactivity of p27 and survivin in chronic gastritis, *H. Pylori *gastritis with/without IM, as well as in IGC lesions. 

In our studied non-neoplastic gastric lesions (chronic gastritis, *H. pylori *gastritis with/without IM), p27 immunoreactivity was detected as cytoplasmic expression, while in IGC lesions; it was detected as nuclear or cytoplasmic staining. The same results were reported by Wen et al., (2012) and Abbastabar et al., (2018) who concluded that p27 has a dual role by acting as an oncoprotein when located in the cytoplasm and as a tumor suppressor when located in the nucleus. Thus cytoplasmic localization of p27 in the non-neoplastic gastric lesions could be an indicator of the progression of these lesions to gastric cancer.

Our data revealed that p27 positivity and expression decreased from controls to *H. pylori *gastritis with a further decrease in IM-associated *H. pylori *gastritis. This is in line with the results of Yu et al., (2001). Eguchi et al., (2004) explained this finding as gastric cells chronically exposed to *H. pylori *develop resistance to apoptosis and were associated with low levels of p27. In contrast to our results, Sougioultzis et al., (2003) observed an increase in epithelial cell apoptosis in *H. pylori *gastritis, which is associated with an increase in p27 expression. These controversial findings could be related to the duration and burden of *H. pylori *infection. 

The current study revealed lower p27 expression in IGC than in control and chronic gastritis. This is in agreement with the results of Yu et al., (2001). Our results confirm the previously published articles reporting a common association between reduced expression of p27 and poor prognosis in many malignancies, including gastric cancer (Sun et al., 2016; Abbastabar et al., 2018) as we found decreased expression of p27 in high-grade and advanced-stage IGCs compared with low-grade and early-stage cancers. This can be explained as gastric cancers with low p27 expression have low levels of apoptosis compared with those cancers expressing higher levels of p27. Data from our studied gastric cancer cases revealed that males had significantly higher p27 positivity and expression than females, this goes with findings of Sun et al., (2016), while Wiksten et al., (2002) found no significant correlation between p27 expression and gender.

According to our findings, p27 expression and localization seem to be important factors for the progression of cancer, as there is an association between lower p27 expression and its cytoplasmic localization which were mainly seen in non-malignant lesions, and gastric cancers with poorer prognosis (high grade and advanced stage). This association may be attributed to the action of p27 as an oncoprotein when it is located in the cytoplasm, leading to progression of cell cycle instead of its primary function as a cell cycle blocker when located in the nucleus (as seen in controls). These data can provide insights into p27 function in tumor progression and therapy.

In the current study, survivin immunostaining was detected mainly as nuclear staining in chronic gastritis, cytoplasmic staining in *H. pylori *gastritis, and nuclear or cytoplasmic staining in IGC cases. This is in agreement with Lins et al., (2016) and Pendely et al., (2018) who reported that cytoplasmic survivin positivity in precancerous lesions was strongly associated with cagA+ *H. pylori *infection and assists in preventing cell apoptosis, whereas nuclear survivin in adenocarcinomas may stimulate cell division frequently observed in advanced malignancy. Consistent with previous reports (Connell et al., 2008; Jones et al., 2008; Nakamura et al., 2004), we found an absence of survivin in control gastric tissue and survivin up-regulation in chronic gastritis, *H. pylori *gastritis with/without IM, and IGCs. 

We detect a highly significant increase in survivin positivity and expression in high-grade and advanced-stage IGCs compared to low-grade and early-stage tumors. This agrees with the results of Wang et al. (2004) and Zhang et al. (2014). We observed that nuclear survivin expression was more frequent in low-grade and early-stage IGCs in comparison to high-grade and advanced-stage IGCs. In the same line, Shintani et al. (2014) found a relationship between the nuclear expression of survivin and well/moderate differentiated gastric tumors and suggested that this nuclear expression is involved in promoting cell proliferation. Meanwhile, in contrast to Shintani et al., (2014) who did not find an association between sex and survivin expression, our data revealed a correlation between survivin positivity and expression with the male sex. 

According to our findings, the absence of survivin expression in controls and the steady rise of its expression from *H. pylori *gastritis to IM to cancer is an important observation that can make survivin an important target for cancer therapeutics. Cytoplasmic localization of survivin was mainly seen in non-malignant lesions and gastric cancers with poorer prognoses (high-grade and advanced-stage). This localization seems to facilitate the progression of these non-neoplastic lesions to gastric cancer.

In addition, we found an inverse correlation between p27 and survivin, without statistical significance. Zhang et al. (2009) suggest that upregulated p27 can downregulate survivin expression and inhibit telomerase activity in gastric carcinoma cells.

The present study has some advantages compared to previous studies. First, it investigated p27 and survivin in non-neoplastic gastric lesions (chronic gastritis, *H. pylori *gastritis with/without IM) and intestinal-type gastric cancer lesions, while most of the previous studies covered only one of these lesions. Second, it evaluated the effect of *H. pylori *infection and IM on the expression of p27 and survivin. However, the main limitation of our study is the relatively small number of studied cases that did not enable us to deeply study the association between cytoplasmic/nuclear localization of both proteins in different studied groups.

In conclusion, our study demonstrated an association between *H. pylori *gastritis with/without IM with low levels (positivity and expression) of p27 and high levels of survivin, as well as, an association between high-grade and advanced-stage IGCs with low p27 and high survivin levels. Taken together, p27 and survivin could be used as diagnostic/prognostic markers in *H. pylori *and IM-associated gastritis as well as in intestinal-type gastric cancer. Besides, both proteins can be used as a targeted therapy in intestinal-type gastric cancer.

## Author Contribution Statement

NSH interpreted all data and wrote the manuscript; ZO, TA, and AB designed the structure and drafted the manuscript; MY, MAA provided specimens and clinical data needed for this study, MM designed and revised the manuscript. All authors read and approved the final manuscript.

## Funding

This work is based upon Research Project, No.114T, supported by TBRI, principal investigator: Assistant Professor Dr. Zeinab Omran.

## Competing Interests

The authors declare that they have no competing interests.

## Data Availability

The datasets analyzed during the current study are available from the corresponding author on reasonable request.
